# Chronic Obstructive Pulmonary Disease Is Associated with Low Levels of Vitamin D

**DOI:** 10.1371/journal.pone.0038934

**Published:** 2012-06-21

**Authors:** Louise Jeanette Pauline Persson, Marianne Aanerud, Pieter Sicco Hiemstra, Jon Andrew Hardie, Per Sigvald Bakke, Tomas Mikal Lind Eagan

**Affiliations:** 1 Department of Thoracic Medicine, Haukeland University Hospital, Bergen, Norway; 2 Institute of Medicine, University of Bergen, Bergen, Norway; 3 Department of Pulmonology, Leiden University Medical Center, Leiden, The Netherlands; University of Tübingen, Germany

## Abstract

**Introduction:**

COPD patients may be at increased risk for vitamin D (25(OH)D) deficiency, but risk factors for deficiency among COPD patients have not been extensively reported.

**Methods:**

Serum 25(OH)D levels were measured by liquid chromatography double mass spectrometry in subjects aged 40–76 years from Western Norway, including 433 COPD patients (GOLD stage II-IV) and 325 controls. Levels <20 ng/mL defined deficiency. Season, sex, age, body mass index (BMI), smoking, GOLD stage, exacerbation frequency, arterial oxygen tension (PaO_2_), respiratory symptoms, depression (CES-D score≥16), comorbidities (Charlson score), treatment for osteoporosis, use of inhaled steroids, and total white blood count were examined for associations with 25(OH)D in both linear and logistic regression models.

**Results:**

COPD patients had an increased risk for vitamin D deficiency compared to controls after adjustment for seasonality, age, smoking and BMI. Variables associated with lower 25(OH)D levels in COPD patients were obesity ( = −6.63), current smoking ( = −4.02), GOLD stage III- IV ( = −4.71, = −5.64), and depression ( = −3.29). Summertime decreased the risk of vitamin D deficiency (OR = 0.22).

**Conclusion:**

COPD was associated with an increased risk of vitamin D deficiency, and important disease characteristics were significantly related to 25(OH)D levels.

## Introduction

The role of vitamin D in preserving skeletal integrity is well known. However, it has become increasingly clear that vitamin D deficiency is associated with increased risk of chronic diseases like cancer, autoimmune diseases, infectious diseases and cardiovascular diseases [1]. There are substantial differences in the prevalence of vitamin D deficiency in various populations; only parts of this variation may be explained by different assays or definition of lower reference limit [2], [3].

Vitamin D deficiency may be a risk factor for respiratory disease. In a report on subjects from a general population in the United States, lower levels of vitamin D were associated with a reduced level of lung function measured by forced expiratory volume in one second (FEV_1_) and forced vital capacity (FVC) [4]. In addition, vitamin D deficiency has been implicated in the risk for developing respiratory infections [5], [6], and increasing asthmatic symptoms or asthma in childhood [7], [8].

Recent studies show that a substantial proportion of patients with chronic obstructive pulmonary disease have deficient vitamin D levels (<20 ng/mL) [9], [10]. In one study of 262 COPD patients from Belgium, FEV_1_, alveolar ventilation (K_CO_) in percent predicted, body mass index (BMI), season of blood sampling and gene variants in the vitamin D binding gene were found to be significantly associated with 25(OH)D levels [9]. However, whether COPD patients actually have a higher prevalence of vitamin D deficiency than the general population is not yet well documented.

In addition, few studies [11] have examined the association between levels of vitamin D and other characteristics of COPD than FEV_1_ such as exacerbation frequency, hypoxemia, comorbidities, and respiratory symptoms. The aim of the current study was to compare levels of vitamin D in COPD patients and healthy controls in a Norwegian population, and to further evaluate the associations between important COPD characteristics to levels of 25(OH)D, including the variables exacerbation frequency, hypoxemia, comorbidities, respiratory symptoms and body mass index.

## Methods

### Ethics Statement

Written information was provided and written consent was obtained prior to inclusion. The regional ethical committee approved the study (Regional committee for medical and health research ethics, Western Norway).

### Study Population

All subjects were recruited from the same geographical area in Western Norway, and participated in the baseline survey of the Bergen COPD cohort Study from 2006 to 2009 at Haukeland University Hospital. The study sample included 433 COPD patients and 325 subjects without COPD, all Caucasians aged 40–76 years.

Causes for exclusion for all subjects were known autoimmune diseases or any active cancer in the last 5 years, but subjects with common comorbidities (including cardiovascular disease and diabetes) were not excluded. A smoking history of ≥10 pack-years and a FEV_1_/FVC ratio <0.7 and FEV_1_<80% predicted were criteria for inclusion. A more detailed description of the recruitment of participants and inclusion/exclusion criteria was previously reported [12].

### Data Sampling

A study physician examined all subjects and performed a structured interview. Briefly, respiratory symptoms including cough with phlegm and dyspnea were reported through a self-completed questionnaire [12]. Information on smoking habits, comorbidities, medication use, and exacerbations was obtained by the study physician. Frequent exacerbations were defined as having ≥2 exacerbations treated with antibiotics and/or oral steroids and/or hospitalization the last 12 months. Arterial blood gases were analysed for arterial oxygen on a Radiometer ABL 520 (Radiometer, Copenhagen, Denmark) within 5 min after sampling by the study physician [13]. An arterial partial oxygen pressure of <8.0 kPa defined hypoxemia. Pulmonary function was measured both pre-and post-inhalation of 0.4 mg salbutamol, on a Viasys Masterscope (Viasys, Hoechberg, Germany) by trained study staff. Body mass index (BMI) was calculated as the weight (kg) divided by the square of height (m^2^), and was categorized as underweight (BMI <18.5), normal (BMI 18.5–24.99), overweight (BMI 25.0–29.99), and obese (BMI 30.0 or more) according to the current World Health Organization (WHO) classification. Comorbidities were categorized using the Charlson Comorbidity Index (CCI) [14]. Depressive symptomatology was measured using the Centre for Epidemiologic Studies Depression Scale (CES-D), where a positive score of 16 or more defined depression [15]. Season was defined as winter (December-March), spring (April-May), summer (June-September), and autumn (October-November).

The study design and data collection has been described in more detail previously [12].

### Laboratory Measurements

Peripheral venous blood was drawn at the baseline visit, and coagulated at room temperature for 30–45 min, followed by centrifugation at 2500×g (15 min 4°C). All serum samples were stored at ≥70°C and thawed immediately before analysis.

Measurement of individual 25(OH)D_3_ and 25(OH)D_2_ levels was performed at the Hormone Laboratory at Haukeland University Hospital using an in-house developed liquid chromatography double mass spectrometry (LC-MS/MS) method [16]. The LC-MS/MS method allowed full distinction between 25(OH)D_3_ and 25(OH)D_2_. The within-day precision (CV) was ≤3.1%, and the between-day precision (CV) was ≤8.7. Only seven individuals had detectible levels of 25(OH)D_2_. Total 25(OH)D was defined as the sum of 25(OH)D_2_ and 25(OH)D_3_.

### Statistical Analyses

Serum concentrations of 25(OH)D were normally distributed. Differences in mean 25(OH)D by bivariate predictors were examined with parametrical tests. We analysed the relation between study category and levels of 25(OH)D both by linear and logistic regression after adjusting for possible confounders: Season, sex, age, BMI, smoking, and comorbidities. A cut off level for vitamin D deficiency were defined as <20 ng/mL [1] for the logistic model. When building the regression models for the levels of vitamin D among COPD patients only, a backward stepwise method was used for both the linear and logistic models. The following variables were included from the start: Age, sex, BMI, disease severity staged accordingly to the global initiative of chronic obstructive lung disease (GOLD), hypoxemia, dyspnea grade III, use of inhaled steroids, comorbidity (Charlson score <2 or ≥2), total white blood cell count, treatment for osteoporosis (yes or no), exacerbation frequency, and CES-D score. Variables remained in the model if their significance level were less than 0.10. After the first run, all excluded variables were reintroduced one at a time and retained in the final model if their significance level were less than 0.10.

A p-value of <0.05 was assumed to indicate statistical significance of observed associations. All analyses were computed with Stata version 10.1 (StataCorp. LP, College Station, TX, USA).

## Results

The characteristics of the study population are shown in Table 1. There were more men than women, both among COPD patients and controls. Difference in mean age between the groups was 4.9 years, with a higher mean in COPD patients (63.5 years). The COPD patients had more comorbidities and 21% had depression as defined by the CES-D score, compared to 6% of the controls. Only one subject was underweighted in the control group (0.5%), versus 8% in the COPD group.

**Table 1:** Baseline characteristics of the study sample, presented as mean±sd for continuous and percentage for categorical variables.

	COPD	Controls	p**
***Subjects, n***	433	325	
***Sex, % females***	40	46	0.11
***Age, years***	63.5±6.9	58.6±9.8	<0.001
***Smoking habits:***			<0.001
Never	0	14	
Ex	56	32	
Current	44	54	
***BMI*** § ***(kg/m^2^)***			<0.001
<18.5	8	0.5	
18.5–24.9	43	39	
25–30	35	45.5	
>30	14	16	
***Comorbidity, Charlsons score***			<0.001
0	0	70	
1	58	22	
2	23	6	
3	12	2	
4	7	0	
***Depression, CES-D score≥16***			<0.001
Yes	21	6	
***Season*** #			<0.001
Winter	16	31	
Spring	21	41	
Summer	56	2	
Autumn	7	26	
***FEV_1_ in percent predicted*** *	49±14	102±10	<0.001
***GOLD status:***			
II (FEV_1_ 50–80)	46		
III (FEV_1_ 30–50)	42		
IV (FEV_1_ 0–30)	12		
***Resting PaO_2_*** §§	9.3±1.2		
***Hypoxemia, % yes***	12		
***Symptoms, % yes***			
Cough with phlegm	59		
Dyspnea (grade III)	44		
***Inhaled steroids, ICS, % yes***	69		
***Exacerbations*** ## ***:***			
<2 last 12 months	83		
≥2 last 12 months	17		
***Total white blood count***	8.1±2.2		
***Vitamin D, 25(OH)D***			0.86
<20 ng/mL	33	34	
≥20 ng/mL	67	66	

§ BMI: body mass index.

* FEV_1_: Forced expiratory volume in 1 s.

## Season was defined as winter (December-March), spring (April-May), summer (June-September), and autumn (October–November).

# Exacerbations requiring either hospitalisation or treatment with oral antibiotics or oral steroids.

§§ PaO_2_: arterial oxygen tension.

** Associations were tested with t-test and Chi-square.

Among COPD patients, 9% had ≥2 exacerbations last year, 69% used inhaled steroids, and overall mean FEV_1_ in percent predicted was 49% (Table 1).

### Factors Associated with 25(OH)D in COPD Patients and Controls - Bivariate Analyses

Concentrations of 25(OH)D among COPD patients and controls for different explanatory variables are shown in Table 2. Unadjusted, there was no significant difference in serum levels of 25(OH)D between COPD patients and controls.

**Table 2:** Serum levels of 25(OH)D in ng/mL, mean±sd, for different potential explanatory variables by subject category.

	COPD	p**	Controls	p**
***25(OH)D, ng/mL***	25.2±10.0		25.0±9.5	
***Sex***		0.58		0.42
Women	25.6±10.4		25.5±9.4	
Men	24.9±9.8		24.6±9.5	
***Age***		0.10		<0.001
40–54	22.5±10.2		20.4±7.9	
55–64	25.0±10.4		27.6±9.4	
>65	26±9.4		28.3±9.0	
***Smoking habits***		<0.01		<0.01
Never			25.9±8.3	
Ex	26.3±9.9		27.5±9.0	
Current	23.6±9.9		23.4±9.7	
***BMI*** * ***(kg/m^2^)***		0.001		0.22
<18.5	21.6±10.0		28.4±0.0	
18.5–24.9	26.0±10.2		25.9±9.4	
25–30	26.5±9.4		25.0±9.4	
>30	21.6±9.7		22.6±10.1	
***Comorbidity, Charlsons score***		0.94		0.31
<2	25.1±10.1		24.9±9.4	
≥2	25.1±9.9		27.0±10.4	
***Depression, CES-D score≥16***		<0.01		
Yes	22.0±10.3			
No	26.0±9.9			
***Season***		<0.001		<0.001
Winter	20.9±9.8		22.0±8.9	
Spring	22.7±9.6		23.7±9.4	
Summer	27.8±9.4		32.0±7.3	
Autumn	20.6±9.5		30.2±8.1	
***GOLD status:***		<0.001		
II (FEV_1_ 50–80)	28.1±9.8			
III (FEV_1_ 30–50)	22.7±9.3			
IV (FEV_1_ 0–30)	21.6±10.0			
***Hypoxaemia (resting*** ***PaO_2_*** § ***<8.0)***		<0.01		
Yes	20.5±8.1			
No	25.9±10.0			
Not measured	24.0±10.7			
***Cough with phlegm***		0.22		
Yes	24.6±10.2			
No	25.±9.7			
***Dyspnea (grade III)***		0.01		
Yes	23.7±9.6			
No	26.3±10.1			
***Inhaled steroids, ICS***		0.05		
Yes	24.5±10.1			
No	26.5±9.5			
***Exacerbations*** # ***:***		0.18		
<2 last 12 months	25.4±9.9			
≥2 last 12 months	23.7±10.5			
***Treatment for osteoporosis***		0.33		
Yes	27.9±10.9			
No	25.0±10.0			

* BMI: body mass index.

# Exacerbations requiring either hospitalisation or treatment with oral antibiotics or oral steroids.

§ PaO_2_: arterial oxygen tension.

** Associations were tested with t-test and ANOVA.

**Figure 1:**
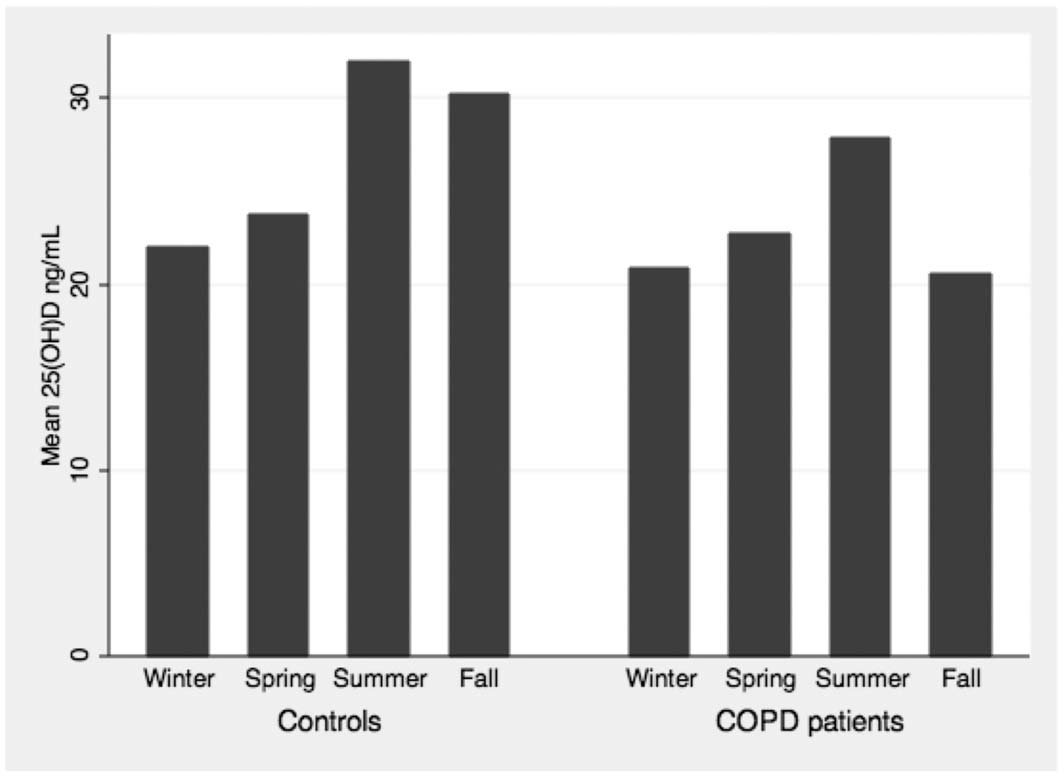
Seasonal variation of measured serum 25(OH)D in controls and COPD patients.

Current smoking and non-summer season were associated with lower levels of serum 25(OH)D in both groups. The importance of season for the measured levels of 25(OH)D is shown in Figure 1.

Among the controls, lower age was associated with lower levels of serum 25(OH)D (Table 2). Among the COPD patients, obesity and underweight, higher GOLD stage, reporting dyspnea, depression, or use of inhaled steroids, were all factors associated with significantly lower levels of serum 25(OH)D (Table 2).

**Figure 2:**
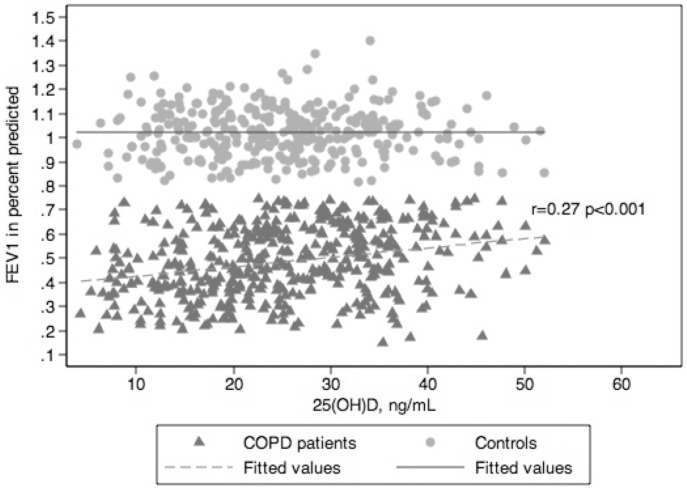
Forced expiratory volume in 1 s (FEV_1_) plotted as a function of serum 25(OH)D levels for COPD patients and controls. The Pearson coefficient (r) is calculated and given in the graph.

Bivariate associations between the continuous variables age, FEV_1_, PaO_2_, and total white blood count with serum levels of 25(OH)D are shown in Table 3. Among the COPD patients, lower FEV_1_ and lower PaO_2_, were associated with lower levels of 25(OH)D (Table 3). The significant correlation between FEV_1_ and 25(OH)D is presented in Figure 2.

**Table 3:** Correlation analysis of continuous variables associated with baseline concentrations of serum 25(OH)D.

	COPD		Controls	
	Pearson’sr	P	Pearson’sr	P
***Age, years***	0.073	0.130	0.375	<0.001
***FEV_1_ in percent predicted***	0.271	<0.001	0.000	0.995
***Resting PaO_2_*** §	0.152	0.003	0.045	0.428
***Total white blood count***	−0.112	0.023	−0.076	0.202

# BMI: Body mass index.

§ PaO_2_: Arterial oxygen tension.

### Difference in Estimated Risk for Vitamin D Deficiency: COPD vs. Controls

COPD status was associated with lower levels of 25(OH)D ( = −4.34, p<0.001) and a more than doubled risk of being categorised as deficient (OR = 2.32, p = 0.001), after adjustment for sex, age, BMI, smoking, comorbidities and season (Table 4).

**Table 4:** Regression coefficients for the relationship between subject status and serum levels of 25(OH)D, adjusted for sex, age, BMI, smoking, comorbidities and season.

	25(OH)D†			25(OH)D* (<20 ng/mL)		
	Coef.	CI	p	OR	CI	p
*Study category*						
**Controls**	0			1		
**COPD subjects**	−4.34	−6.31,−2.37)	p<0.001	2.32	(1.43,3.75)	p = 0.001

† Linear regression model.

* Logistic regression model.

### Factors Associated with 25(OH)D in COPD Patients - Multivariate Analyses

After adjustment, GOLD stage, obesity, smoking, and season remained associated with both mean levels of 25(OH)D and total deficiency (Table 5). Higher GOLD stage was associated both with lower mean levels of 25(OH)D and increased risk for deficiency. In summertime compared to wintertime, mean levels were higher and the risk of deficiency was lower. Current smokers had lower levels of 25(OH)D, and the risk of deficiency was significantly higher. Patients with obesity had lower 25(OH)D levels and a significantly increased risk for deficiency.

**Table 5:** Coefficients from multiple linear regression and logistic regression models, showing the relationship between baseline predictors and serum levels of 25(OH)D in COPD patients.

	25(OH)D			25(OH)D <20 ng/mL		
	Coef.	CI	p	OR	CI	P
***Smoking habits***						
Ex	0			1		
Current	−4.02	−6.05, −1.99)	<0.001	3.15	(1.81, 5.47)	<0.001
***BMI*** * ***(kg/m^2^)***	−0.37	−0.57, −0.17)	<0.001	1.01	(1.02, 1.14)	0.005
<18.5	−2.85	−6.81, 1.11)	0.16	1.44	(0.52, 3.97)	0.48
18.5–24.9	0			1		
25–30	−1.52	−3.77, 0.74)	0.19	1.08	(0.57, 2.05)	0.8
>30	−6.63	−9.54, −3.72)	<0.001	4.26	(1.97, 9.19)	<0.001
***Season*** #						
Winter	0			1		
Spring	3.12	−0.03, 6.26)	0.052	0.72	(0.33, 1.58)	0.42
Summer	6.99	(4.26, 9.73)	<0.001	0.22	(0.11, 0.46)	<0.001
Autumn	2.71	−1.78, 7.19)	0.24	0.57	(0.19, 1.71)	0.31
***GOLD status:***						
II (FEV_1_ 50–80% predicted)	0			1		
III (FEV_1_ 30–50% predicted)	−4.71	−6.86, −2.56)	<0.001	3.19	(1.72, 5.91)	<0.001
IV (FEV_1_ 0–30% predicted)	−5.64	−9.02, −2.25)	0.001	7.13	(2.88, 17.64)	<0.001
***Depression, CES-D score≥16***						
Yes	−3.29	−5.76, −0.81)	0.009	1.74	(0.92, 3.31)	0.089
No	0			1		

* For both the linear and logistic regression models a backward stepwise procedure was used with the following variables included at start: Age, sex, GOLD status, hypoxemia (resting PaO_2_<8), dyspnea (grade III), inhaled steroids, ICS (yes or no), *body mass index (BMI), comorbidity (Charlsons score <2 or ≥2), total white blood count, treatment for osteoporosis (yes or no), depression (CES-D score≥16) and exacerbation frequency (≥2 last year; yes or no).

# Season was defined as winter (December–March), spring (April–May), summer (June–September), and autumn (October–November).

Depression was associated with lower levels of vitamin D (Table 5), but only in the linear regression model. Although hypoxemia and dyspnea were related to 25(OH)D in the bivariate analyses, these relationships were not statistically significant in the multivariate analyses.

## Discussion

The prevalence of vitamin D deficiency was high in both COPD patients and controls. When correcting for season, age, smoking, comorbidities, and BMI, the estimated risk for vitamin D deficiency was shown to be higher in COPD patients compared to controls. In COPD patients, there was a significant association between vitamin D levels and FEV_1_ in percent predicted, whereas hypoxemia and exacerbation frequency the last year were not associated with vitamin D levels. Previous studies have described vitamin D deficiency as a common phenomenon in elderly populations [2], [3]. In line with these observations, also in our study population we observed a high prevalence of vitamin D deficiency; 34% in the controls and 20%, 43% and 55% in the patients with GOLD stage II-IV respectively. Although these are high numbers, the prevalence was lower than what has been reported in two previous studies [9], [10]. A possible explanation for this difference could be a high year-around consumption of fish and cod liver oil in Norway, which naturally contains 25(OH)D_3_. Another explanation could be differences between study samples in regard to COPD severity.

Season was a strong predictor of 25(OH)D levels, and relatively more patients than controls were examined during summer in our study sample. After adjustment for season, the real difference in risk for vitamin D deficiency between COPD patients and healthy controls became apparent. There are several potential explanations for the increased risk for vitamin D deficiency in COPD patients. The COPD patients have physical impairments leading to inactivity and less time spent outdoors. Current smoking is a known risk factor for vitamin D deficiency [17], but smoking was adjusted for in the multivariable analyses. However, COPD patients may have accelerated skin ageing due to smoking, renal dysfunction, less fat tissue due to wasting, and treatment with glucocorticoids, all factors that can affect vitamin D synthesis, storage or catabolism [1].

In the NHANES III study, Black et al. found a dose- response relationship between 25(OH)D and both FEV_1_ and FVC, but no correlation with airway obstruction defined as a change in FEV_1_/FVC ratio [4]. However, in a recent study on COPD patients by Janssens et al. [9], a strong relationship was found between GOLD stage and vitamin D deficiency suggesting a relationship with airway obstruction. A similar association between 25(OH)D and FEV_1_ has been reported in adults with asthma [18]. Our study, which included more COPD patients and where we adjusted for potential confounders not assessed by Janssens et al., shows a nearly identical relationship between airways obstruction and vitamin D levels. Together these studies provide strong evidence for this relationship, although the causality of this association remains to be established.

What are the possible factors that may explain the association between lung function and vitamin D levels? Several extra-renal sites, including cells of the adaptive immune system, express the vitamin D receptor and the enzyme, 1-hydroxylase, which converts circulating 25(OH)D into the biologically active form 1,25(OH)_2_D [1]. Both the innate and adaptive immune systems have been implicated as important elements in the pathogenesis of COPD [19]. Interestingly, vitamin D has been shown to be an important regulator of both elements of the immune system. Vitamin D has been shown to affect dendritic cell maturation [20], T-cell activation and proliferation [21], and Th1 T-cell development [22]. In addition, vitamin D deficiency has been shown to be associated with increased susceptibility to respiratory infections [5], [6], which may in part be explained by the ability of 1,25(OH)_2_D to increase expression of antimicrobial peptides [23], and to decrease the expression of pro-inflammatory cytokines and chemokines by e. g. airway epithelial cells [24]. Thus, low local 1,25(OH)_ 2_D levels could potentially contribute to inflammation and susceptibility to infections, which may lead to an increased rate of FEV_1_ decline [25]. In addition to its effects on immunity and inflammation, vitamin D also has been shown to directly affect processes involved in tissue remodelling such as fibroblast proliferation and collagen synthesis [26], and modulation of matrix metalloproteinase (MMP) levels [27]. Moreover, undiagnosed osteoporosis leading to vertebral compressions may lead to loss in height, reduced rib cage mobility and a decline in pulmonary function [28].

Adiposity has in previous studies been a significant predictor of low levels of vitamin D in both COPD patients and subjects without COPD [9], [29]. As expected, we found that obesity was negatively associated with levels of vitamin D and deficiency in COPD patients. A suggested explanation for obesity-associated vitamin D insufficiency, is a decreased bioavailability of 25(OH)D_3_ when deposited in body fat compartments [29].

Several studies have found an increased prevalence of vitamin D deficiency in subjects with depression [30], [31] or depressive symptoms [32]. Patients with depression typically spend less time outdoors and exhibit less physical activity. These factors are also associated with more severe COPD, and could confound a relationship between vitamin D deficiency and COPD. In the current study, the addition of depression to the multivariate models did not change the increased risk of vitamin D deficiency in COPD patients compared to controls, nor did it change the observed relationship between vitamin D and FEV_1_.

However, our data showed a significant independent association between depression and lower levels of vitamin D in COPD patients. To our knowledge this has not been previously reported in COPD patients. Whether this is caused by factors such as less physical activity, less time spent outdoors and change in diet we cannot say from the current study. Longitudinal studies, taking these potential confounders into account, are necessary to address this important issue.

Several studies demonstrate that vitamin D plays a role in susceptibility to airway infections [5], [6]. One explanation for this is the already mentioned role of vitamin D in the innate immune response, including its ability to induce the production of antimicrobial peptides [23]. In Costa Rican children with asthma, the number of hospitalizations following exacerbation showed an inverse relationship with levels of 25(OH)D [7]. The NHANES III study reported an inverse dose-response relationship between levels of 25(OH)D and upper respiratory tract infection in individuals with asthma, and a similar trend was seen for COPD patients although not significant after adjustment [5]. In the current study, we did not find an association with self-reported exacerbation frequency in the previous 12 months and levels of 25(OH)D. It might be that our indicator variable for exacerbation frequency was not precise enough for the outcome, or that the number of COPD patients that had experienced ≥2 exacerbations the last year was too low (9%). However, the results are in line with a recent one-year follow-up study, where baseline vitamin D levels failed to predict exacerbations rates in a patient sample from a randomized controlled trial (RCT) on Azithromycin [11]. Also, the first RCT on vitamin D supplements and subsequent COPD exacerbations was recently published, failing to show a positive effect of supplements [33]. It is worth noting that due to the small patient sample of 182 patients in this RCT [33], a rather large effect of vitamin D would be necessary in order to produce a significant difference between the groups.

These three studies together imply either that the relationship between vitamin D and COPD exacerbations is weaker or may be more complicated than hypothesized. More and larger longitudinal studies are needed to elucidate this issue.

The strength of the current study was its large sample size with a large number of baseline data recorded and accurate measurement of vitamin D levels using state-of-the-art methodology (LC-MS/MS). However, there are some methodological issues to consider. First, we must point out that this is a cross-sectional study and therefore inference of cause and effect is not possible. Second, although diet might be a minimal contributor to vitamin D status [34], lack of data on dietary intake or use of vitamin D preparations is a limitation of our study. It is rare in Norway to take vitamin D supplements, and we find no reason to assume a difference in use of supplements within our study population compared to the general population. The prevalence of subjects with detectable levels of 25(OH)D_2_ was low (<0.01%), which is consistent with the fact that in Norway 25(OH)D_3_ is used almost exclusively is used as fortification and supplements [16].

Third, there was a significant difference in age between COPD patients and controls, where the COPD patients were on average 5 years older than the controls. Among the controls, lower age was significantly associated with lower levels of vitamin D, whereas no association was found between age and vitamin D levels among the COPD patients. Thus, it was important to adjust for age in the multivariate models, to avoid a false negative association between COPD status and vitamin D deficiency, due to confounding by a younger control group.

In conclusion, COPD patients had an increased risk for having vitamin D deficiency, and the relationship between lung function and systemic levels of vitamin D was almost linear even after adjustment for a large number of known and potential confounders. Future longitudinal studies are warranted to assess the predictive effect of levels of vitamin D on decline in lung function, risk for depression, exacerbation frequency, and changes in body composition in patients with COPD.
